# The critical role of dysregulated autophagy in the progression of diabetic kidney disease

**DOI:** 10.3389/fphar.2022.977410

**Published:** 2022-08-25

**Authors:** Ziwei Zhang, Yuting Sun, Jiaojiao Xue, De Jin, Xiangyan Li, Daqing Zhao, Fengmei Lian, Wenxiu Qi, Xiaolin Tong

**Affiliations:** ^1^ College of Traditional Chinese Medicine, Changchun University of Chinese Medicine, Changchun, China; ^2^ Department of Endocrinology, Guang’anmen Hospital, China Academy of Chinese Medical Sciences, Beijing, China; ^3^ Hangzhou Hospital of Traditional Chinese Medicine, Hangzhou, China; ^4^ Northeast Asia Research Institute of Traditional Chinese Medicine, Key Laboratory of Active Substances and Biological Mechanisms of Ginseng Efficacy, Ministry of Education, Jilin Provincial Key Laboratory of Biomacromolecules of Chinese Medicine, Changchun University of Chinese Medicine, Changchun, China; ^5^ Guang’anmen Hospital, China Academy of Chinese Medical Sciences, Beijing, China; ^6^ Institute of Metabolic Diseases, Guang’anmen Hospital, China Academy of Chinese Medical Sciences, Beijing, China

**Keywords:** diabetic kidney disease, autophagy, autophagosome, lysosome, podocytes, renal tubular epithelial cells

## Abstract

Diabetic kidney disease (DKD) is one of the major public health problems in society today. It is a renal complication caused by diabetes mellitus with predominantly microangiopathy and is a major cause of end-stage renal disease (ESRD). Autophagy is a metabolic pathway for the intracellular degradation of cytoplasmic products and damaged organelles and plays a vital role in maintaining homeostasis and function of the renal cells. The dysregulation of autophagy in the hyperglycaemic state of diabetes mellitus can lead to the progression of DKD, and the activation or restoration of autophagy through drugs is beneficial to the recovery of renal function. This review summarizes the physiological process of autophagy, illustrates the close link between DKD and autophagy, and discusses the effects of drugs on autophagy and the signaling pathways involved from the perspective of podocytes, renal tubular epithelial cells, and mesangial cells, in the hope that this will be useful for clinical treatment.

## 1 Introduction

Diabetes mellitus (DM) is a common and prevalent disease in recent years, characterized by chronic hyperglycaemia (Davidson et al., 2015). Diabetic kidney disease (DKD) is a serious microvascular complication of DM, which may present with a glomerular hyperfiltration state and microalbuminuria in the early stages, with disease progression to oedema and massive proteinuria and severe impairment of renal function (Reidy et al., 2014). According to reports, the number of people with DM will increase to 592 million by 2035 and about 35–40% of them will eventually progress to DKD, which is a major cause of end-stage renal disease (ESRD) and brings heavy psychological and financial burden to families and society (Cao and Cooper, 2011; Shi and Hu, 2014). Traditional treatments have focused on smoking cessation, diet control, weight loss, low protein diets, and lowering blood pressure and glucose and lipids to cope with the range of pathological changes caused by hyperglycemia (Lin et al., 2018). These methods provide some relief from DKD, but they cannot stop the progression of DKD to chronic kidney disease (McGrath and Edi, 2019). Therefore, new treatments for DKD are an urgent problem to be solved.

Autophagy is a conserved intracellular pathway that maintains cellular homeostasis by forming autolysosomes to degrade cytoplasmic components (Kitada and Koya, 2021). The term “autophagy” was first coined by Christian de Duve in 1967 as “self-eating” (Deter and De Duve, 1967). The degradation of mitochondria and other cellular components following glucagon stimulation in rats was observed and it was suggested that the autophagic process requires induction and activation of cyclic adenosine monophosphate (AMP) (Deter and De Duve, 1967). In recent years, autophagy has been intensively studied by many researchers and is considered to be a strictly cellular physiological regulatory process widely present in mammalian cells (Klionsky et al., 2021). And the molecular mechanisms associated with autophagy have been studied and discovered in yeast (Takeshige et al., 1992; Suzuki et al., 2001; Kim et al., 2002).

A growing number of studies have shown that the development of DKD is associated with a defect in autophagy in kidney (Deretic, 2021). Restoring autophagy can improve renal function, reduce the occurrence of oxidative stress, inhibit renal inflammation and fibrosis, and slow the progression of DKD, making it to be another important target for the treatment of DKD (Yang et al., 2018). But the literatures on the relationship between drugs and autophagy in DKD is currently poorly organized. Therefore, this review will discuss the important role of autophagy in DKD in terms of the two processes of autophagosome formation and autolysosome formation, in the hope of laying a theoretical foundation for the clinical treatment of DKD.

## 2 Autophagy

Three types of autophagy exist in mammalian cells: microautophagy, macroautophagy and chaperone-mediated autophagy (Li and Nakatogawa, 2022). Microautophagy is when proteins become trapped in vesicles formed by lysosomes and are subsequently degraded in the vacuolar lumen of lysosomes (Li and Nakatogawa, 2022). Chaperone-mediated autophagy is a highly specific form of protein catabolism mediated by the heat shock cognate 70 (Hsc70), which targets the lysosomal membrane directly into the lumen of the lysosome (Cuervo and Wong, 2014). In addition, mitophagy is a specific type of autophagy with a high degree of selectivity in its classification, which responds to organelle damage and pathogen invasion, and remove mitochondrial toxicity products (Bravo-San Pedro et al., 2017). This review focuses on the role of macroautophagy (hereafter referred to as autophagy) in DKD.

Autophagy is a highly dynamic process that includes several stages of initiation, extension, closure and degradation (Antonioli et al., 2017). Autophagy is primarily regulated by autophagy-associated proteins (ATG), which are encoded and modified by a set of highly conserved ATG genes (Levine and Kroemer, 2019). The mammalian target of rapamycin (mTOR) and AMP-activated kinase (AMPK) are two key molecules in the process of autophagy (Inoki et al., 2012). AMPK acts as an energy receptor and is activated when the AMP/ATP ratio increases in the absence of intracellular energy, while mTOR negatively regulates the process of AMPK activation and inhibits the onset of autophagy (Li and Chen, 2019). mTOR mainly works in two multi-protein complexes: mTORC1 and mTORC2 (Fantus et al., 2016). mTOR inhibits the activation of autophagy and promotes protein and nucleotide synthesis to facilitate cell growth, proliferation and differentiation, and also acts as an amino acid receptor (Kim and Guan, 2019). When cells are stimulated by amino acid signals, mTORC1 phosphorylates the downstream ribosomal protein S6 kinase (S6K) and the eukaryotic translation initiation factor 4E-binding protein 1 (4E-BP1) to promote protein synthesis (Yuan J. et al., 2020).

**FIGURE 1:**
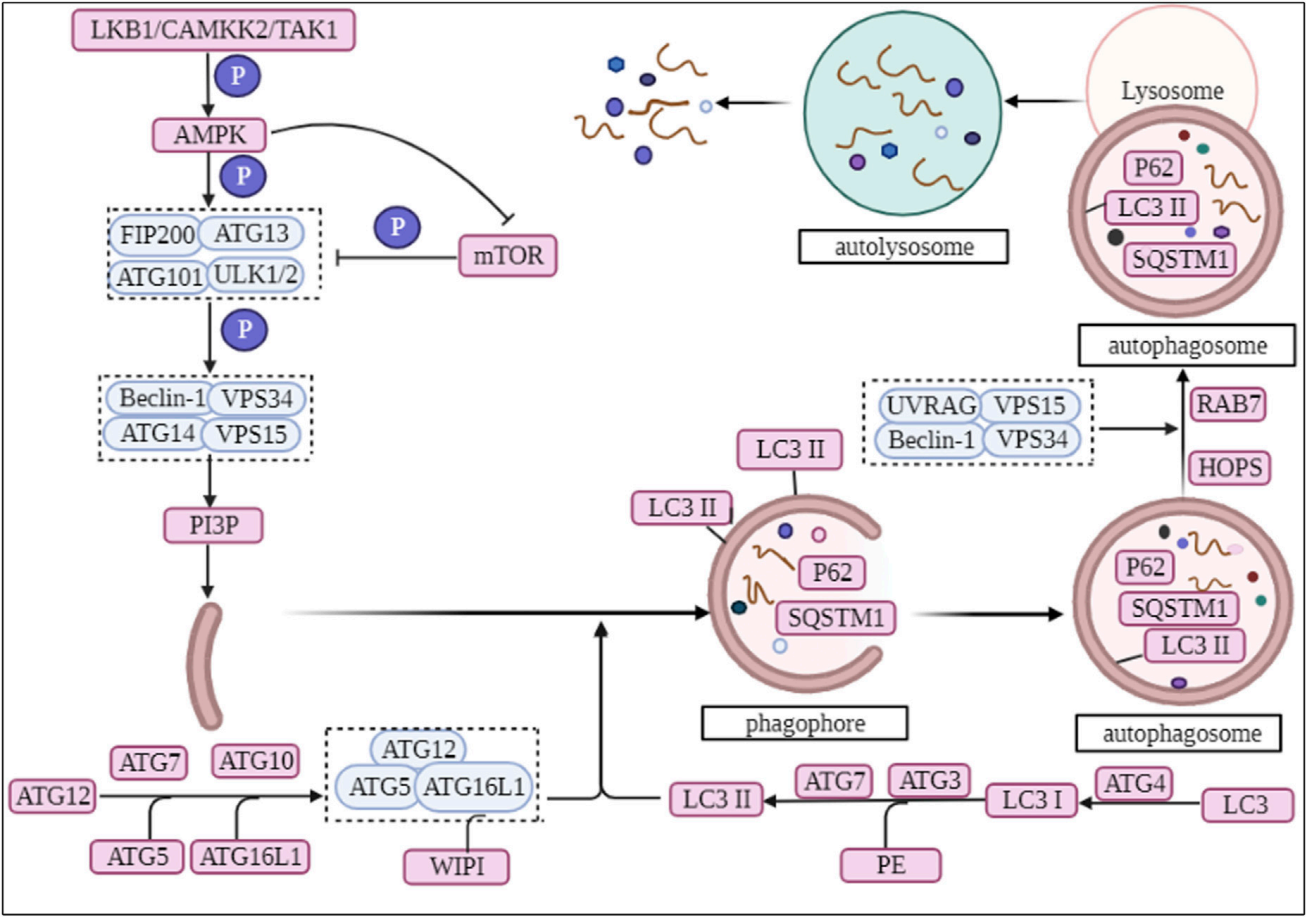
The biological process of autophagy.

In the regulation of autophagy, AMPK is regulated by upstream calcium/calmodulin-dependent protein kinase 2 (CAMKK2, also called CAMKKβ) and serine/threonine-protein kinase stk11 (LKB1), while suppressing the expression of mTORC1 (Shaw et al., 2004; Hawley et al., 2005). Subsequently, AMPK phosphorylates the downstream Unc-51-like (ULK)1/2 complex (Alers et al., 2012). In mammals, ATG101 is an important component of the ULK1 complex and these components that make up ULK1 are responsible for inducing conformational changes to activate the ULK1 complex (Mercer et al., 2009; Lin and Hurley, 2016). The activation of ULK1 phosphorylates the downstream class III phosphatidylinositol three kinase (PI3K) complex (also known as VPS34 complex I), whose main target is Beclin-1. The complex is composed of the core protein PI3K, the autophagy-specific cofactor Beclin-1 and other important factors (Russell et al., 2013; Baskaran et al., 2014; Levine et al., 2015). Subsequently, the PI3K complex promotes the production of phosphatidylinositol-3-phosphate (PI3P), which recruits autophagy factors required for autophagosome formation and promotes the formation of omegasomes (omegasomes are derived from the part of the endoplasmic reticulum (ER) that is connected to other organelles) (Axe et al., 2008; Hayashi-Nishino et al., 2009; Lamb et al., 2013). In mammals, the isolation membrane is supported by ER and may be connected by membrane tubules (Hayashi-Nishino et al., 2009; Ylä-Anttila et al., 2009). The simultaneous recruitment of the effect factors double FYVE zinc finger domain containing protein 1 (DCFP1) and WD repeat domain phosphoinositide-interacting protein (WIPI) contribute to the continuous extension of the phagophore (Dooley et al., 2014; Rogov et al., 2014). ATG2 and the transmembrane protein ATG9 are thought to be autophagosomal intermembrane and interfollicular lipid transport proteins, involved in the expansion of the phagophore (Noda, 2021) (Figure 1).

Subsequently, ATG16L1 acts as an E3 ligase, promotes ATG8 lipidation and successively binds to ATG5 and ATG12, forming an ATG12-ATG5-ATG16L1 ubiquitin-like binding complex (Popelka et al., 2021). Under starvation-induced conditions, WIPI binds to ATG16L1 and recruits the ATG12-ATG5-ATG16L1 complex allowing it to aggregate to the omegasome site of the phagophore (Dooley et al., 2014). Microtubule-associated proteins 1A/1B light chain 3B (LC3), a homologue of mammalian ATG8, is hydrolytically cleaved by ATG4 protein to produce LC3I, which is then activated by ATG7 and ATG3, followed by covalent binding of LC3I by phosphatidylethanolamine, and finally conversion of LC3 to LC3II (Sou et al., 2006; Glick et al., 2010). Researchers have identified the presence of LC3II on both the internal and external surfaces of phagophore, where it plays a role in membrane fusion and sorting of degraded cargo (Glick et al., 2010; Rogov et al., 2014), as well as controlling the size of autophagosomes (Nakatogawa et al., 2007) (Figure 1).

Finally, autophagosomes transports cargo into lysosomes through fusion with lysosomes to form autolysosome (Mizushima and Komatsu, 2011). The fusion of autophagosomes with lysosomes is regulated by the VPS34 complex II, which differs from the PI3K complex in that the subunit ATG14 has been replaced with UV radiation resistance associated (UVRAG) (Lamb et al., 2013). Ras-related protein 7 (Rab7) and ADP-ribosylation factor-like GTPase Arl8 play a key role in the localization of autophagosomes and lysosomes during the fusion process (Pu et al., 2016). They bind several motor junction proteins that link autophagosomes and lysosomes to cytoskeleton-associated motor proteins that allow autophagosomes and lysosomes to move along microtubules (Garg et al., 2011; Pu et al., 2016). Homotypic fusion and vacuole protein sorting (multisubunit complex HOPS) are essential for autolysosome formation, which tether mobile vesicles along the cytoskeleton (Bröcker et al., 2012). Subsequently, the lipid component of autophagosomes regulates autophagosome-lysosome fusion, where the amount and ratio of PI(3)P and PI(3, 5)P2 regulates the formation of autolysosomes (Hasegawa et al., 2016). Finally, the lysosomes release the degraded products extracellularly in the form of cytosolic spit, facilitating the recycling of the degradation products (Buratta et al., 2020) (Figure 1).

Many clinical therapeutic agents for DKD have been shown to activate autophagy and influence the progression of DKD through different signaling pathways, including the classical drug for lowering sugar - metformin, the classic mTOR target inhibitor - rapamycin, and emerging therapeutic agents such as sodium-glucose cotransporter (SGLT)2 inhibitors and glucagon-like peptide 1 (GLP-1) activators (Gonzalez et al., 2021). In addition, in the ongoing research on Chinese herbal extracts, active ingredients such as resveratrol, hispidulin and berberine have shown renoprotective effects based on the induction of autophagy in kidney (which we will discuss in more detail in the following subsections) (Wang et al., 2019c).

## 3 DKD

The traditional pathogenesis of DKD is mainly associated with hyperglycemia: elevated blood glucose promotes metabolic disturbances in DKD, like activating oxidative stress in the kidney, contributing to excessive production of reactive oxygen species (ROS) and reducing activity of antioxidant substances (Fang et al., 2012; Tuttle, 2017). The protein kinase C-α and polyol and hexosamine pathways are activated, upregulating ROS production and increasing the expression of the associated inflammatory factors interleukin (IL) 6, monocyte chemoattractant protein-1, tumour necrosis factor alpha and intercellular adhesion molecule-1 in DKD, accelerating the progress of DKD (Wu X. Q. et al., 2021; Dusabimana et al., 2021; Jha et al., 2022). In addition, imbalances in lipid metabolism, including increased lipid uptake or synthesis, are also important triggers for the development of DKD (Mitrofanova et al., 2021). In a hyperglycaemic environment, small arteries have abnormal systolic-diastolic function, endothelin-1secretion is increased, promoting vascular dysfunction (Potenza et al., 2009; Tuttle, 2017). At the same time, genetic factors and activation of the renin-angiotensin-aldosterone system also contribute to the development of DKD (Vegter et al., 2012; Kato and Natarajan, 2019).

Controlling blood glucose, lipids and blood pressure are common methods in the treatment of DKD (Doshi and Friedman, 2017). Moreover, lifestyle changes such as: limiting protein and sodium intake, exercise, weight loss and smoking cessation, which can slow the progression of DKD and reduce the risk of cardiovascular events (Lin et al., 2018).

Proteinuria is a key indicator in the diagnosis of DKD and an important factor in the development of DKD (Colhoun and Marcovecchio, 2018). Coresh J et al. showed that changes in proteinuria were consistently associated with subsequent risk of ESRD through a cohort study (Coresh et al., 2019). Results from an 11-years observational cohort study from Japan showed that non-albuminuric DKD had a lower risk of mortality, cardiovascular events or abnormal kidney function compared with other DKD phenotypes (Yokoyama et al., 2020). Blood passes through the glomerular capillary network, passing through the glomerular filtration barrier to allow primary urine to enter the Bowman’s space, which is then reabsorbed by the renal tubules and finally excreted as a virtually protein-free urine (Mora-Fernández et al., 2014). Here, researchers have introduced an important concept: the glomerular filtration barrier (GFB), the disruption of which is an important cause of DKD proteinuria (Pavenstädt et al., 2003). It is a physiological barrier structure composed of glomerular endothelium, glomerular basement membrane (GBM) and podocytes and slit-diaphragm (SD) from the inside out, allowing a highly selective ultrafiltration of plasma components (Mora-Fernández et al., 2014; Lin and Susztak, 2016). The glycocalyx of the glomerular endothelium has specific molecular and charge properties and therefore constitutes a charge barrier for the glomerulus (Weinbaum et al., 2007). Thus, when endothelial cell dysfunction, GBM and podocyte damage occur, these lead to the production of proteinuria followed by glomerulosclerosis and a decrease in glomerular filtration (Jefferson et al., 2008).

The foot-process is an essential component of the podocyte, with interlocking foot-process wrapping around the capillary bulb to form filtering slits that bridge with the electron-dense membrane-like structure SD (Huber and Benzing, 2005). High glucose-induced ROS production during DKD promotes podocyte detachment and apoptosis (Susztak et al., 2006). The non-renewable nature of the podocyte leads to a reduction in the number of podocytes, with subsequent disruption of the normal structure of the GFB and podocytes, weakened the connections between podocytes and finally the leakage of non-selective proteins (Susztak et al., 2006; Jefferson et al., 2008). Downregulation of nephrin expression, a key component of SD in the DKD environment, results in abnormal podocyte function (Doublier et al., 2003). The imbalance in activation of the small GTP-binding proteins Rac1 and Cdc42 (Cdc42 regulates the podocyte actin cytoskeleton), validates the link between podocyte injury and proteinuria as well as glomerulosclerosis at another level (Blattner et al., 2013). Autophagy deficiency is also an important participant in podocyte damage and will be discussed in the next section of the article.

In the course of DKD, damage to renal tubular epithelial cells has a greater correlation with kidney dysfunction (Carney, 2016). A series of inflammatory and oxidative stress responses generated by the massive protein leakage in DKD patients leads to morphological and functional dysfunction of the renal tubules, with epithelial mesenchymal transformation and cell detachment as well as apoptosis, ultimately leading to fibrosis (Chen et al., 2020). In addition to this, pathological changes in DKD glomeruli are often accompanied by proliferation and expansion of the mesangial matrix, and activation of mesangial cells driven by factors such as hyperglycaemia and dyslipidaemia, accelerating glomerulosclerosis (Ebefors et al., 2021).

By reviewing the literatures, We have learned that advanced glycation end products (AGEs) are irreversible metabolites produced by hyperglycaemia and AGEs contribute to the production of ROS and mediate inflammatory processes (Matoba et al., 2020; Wu X. Q. et al., 2021; Jha et al., 2022). However, AGEs can be degraded by autophagy and inhibit the inflammatory response (Takahashi et al., 2017). A study of autophagy knockout followed by streptozotocin (STZ) induced DM showed severe microalbuminuria, podocyte and endothelial cell damage in the model mice compared to the control mice (Lenoir et al., 2015). And the renal pathological findings also showed extensive mesangium expansion and glomerulosclerosis, thus demonstrating the close relationship between DKD and autophagy (Lenoir et al., 2015).

## 4. DKD and autophagy

As mentioned previously, we have discussed the biological process of autophagy and its function in cellular metabolism (Kitada and Koya, 2021); and have argued for a close link between autophagy and DKD by reviewing the literatures (Lenoir et al., 2015). Therefore, we will explore how do drugs target autophagy for the treatment of DKD based on two processes, one is autophagosome formation and another is autolysosome formation, in the hope of enhancing the understanding of the relationship between autophagy and DKD.

### 4.1 The role of autophagosome formation in DKD

#### 4.1.1 Role of autophagy in podocytes

**FIGURE 2:**
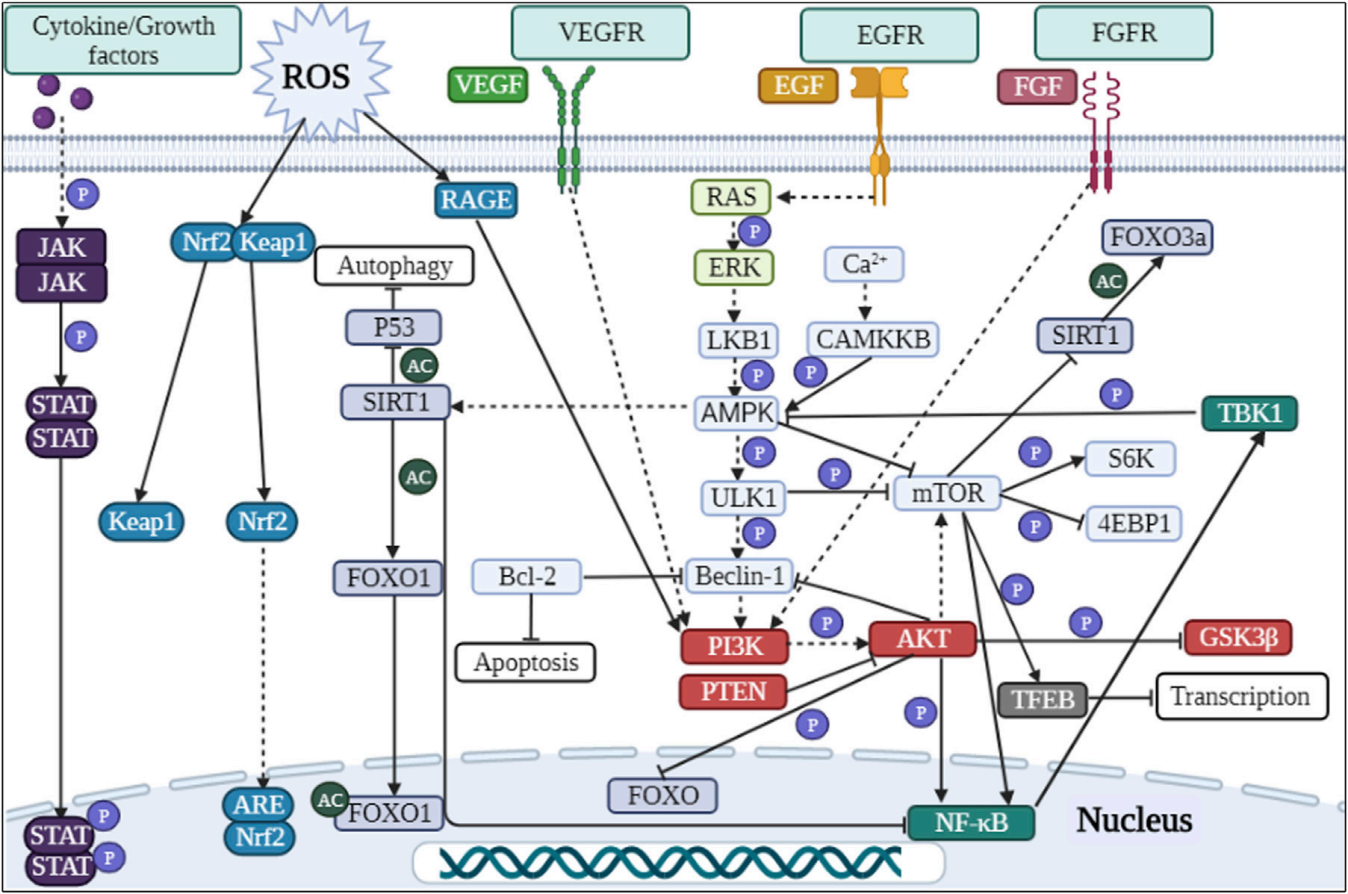
Signaling pathways involved in autophagy regulation in DKD.

In diabetic mice and murine podocyte cell (MPC-5) models, ruxolitinib or wogonin affected autophagy and delayed the progression of DKD through JAK/STAT and Bcl-2-mediated pathways (Chen D. et al., 2021; Liu et al., 2022). In another experimental study, overexpression of KLF4 (a zinc finger-containing transcription factor) showed that KLF4 could play a nephroprotective role by regulating the mTOR/S6K pathway to activate autophagy (Gong et al., 2019). Paecilomyces cicadae-fermented radix astragali played a role in activating autophagy by inhibiting the PI3K/AKT/mTOR signaling pathway, thereby alleviating the progression of DKD (Yang F. et al., 2020). In two experiments using db/db mice and MPC-5 cells as the primary targets, the calcium-sensitive receptor type II agonist cinacalcet worked by activating the CaMKKβ-LKB1-AMPK pathway, while the exosome generated by adipose-derived stem cells achieved therapeutic effects by inhibiting the Smad1/mTOR pathway (Lim et al., 2018; Jin et al., 2019). Similarly, sarsaapogenin (the main active ingredient in Anemarrhena asphodeloides Bunge) and spermine, both of which promoted autophagy by targeting GSK3β and AMPK/mTOR, respectively (Li et al., 2021; Zhang et al., 2021). In a podocyte model induced by high glucose, celastrol restored autophagy through an HO-1-mediated pathway while alleviating the inflammation and damage induced by high glucose in podocytes (Zhan et al., 2018). At the same time, in two additional experiments with high glucose induced podocytes, the expression of G-protein-coupled receptor 43 (GPR43) or sperm-associated antigen 5 (SPAG5), both of which promoted autophagy via the ERK/EGR1 and SPAG5/AKT/mTOR signaling pathways, were inhibited by GPR43 gene knockout or SPAG5 gene silencing, it could be used as potential therapeutic targets for DKD (Xu et al., 2020; Lu et al., 2022). In animal and cellular models of DKD, Astragaloside IV (AS-IV) induced autophagy by activating the AMPK pathway (Guo et al., 2017). In KK-Ay mice and podocyte models, treatment with apelin or AS-IV was administered and both were shown to play an important role in alleviating DKD by regulating the ERK/AKT/mTOR and SIRT1-NF-κB P65 signaling pathways (Liu et al., 2017; Wang X. et al., 2019). Interestingly, the use of hispidulin or berberine in DKD cell models, both of which promoted autophagy by inhibiting mTOR-related pathways (Wu et al., 2018; Li et al., 2020). Inhibition of mTOR and TFEB affected the formation and conversion of autophagy in DKD mice and cellular models, respectively (Zhao X. et al., 2018). Moreover, many researches have also revealed the effects of protein tyrosine phosphatase 1B, resveratrol, ursodeoxycholic acid and 4-phenylbutyrate as well as histone deacetylase 4 on the autophagic process of podocytes in DKD models (Wang et al., 2014; Cao et al., 2016; Huang et al., 2017; Ito et al., 2017). Therefore, autophagy affects cell metabolism in podocytes through mTOR, AMPK, PI3K and other related pathways, inhibits inflammation and kidney damage, and delays the progress of DKD (Figure 2; Tables 1-3, Table.5).

**TABLE 1:** The role of autophagosome formation in DKD (drugs with clinical trials).

Signaling pathway	Processing method	Modeling method (animal/cell)	Conclusion	References
AMPK-mediated signaling pathway	Empagliflozin	STZ induced C57BL/6J mice; HG induced human renal proximal tubular cells	Empagliflozin could reverse mitochondrial dynamics and autophagy so as to play the role of renal protection	Lee et al. (2019)
mTOR/P70S6K/4EBP1 signaling pathway	Berberine	HG-induced podocytes	Berberine activated podocyte autophagy by inhibiting the mTOR/P70S6K/4EBP1 signaling pathway, thereby alleviating podocyte apoptosis	Li et al. (2020)
Ca-CAMKK2-AMPK signaling pathway	Paricalcitol (an activated vitamin D analog)	STZ induced VDR-KO mice; HG induced HK-2 cells	VD-VDR could restore defective autophagy in the kidney of STZ-induced diabetic mice, which could be attributed to the activation of the Ca-CAMKK2-AMPK pathway in renal tubular epithelial cells	Li et al. (2022)
AMPK/SIRT1-FOXO1 signaling pathway	Metformin	HFD and STZ induced SD rats; HG cultured RMCs	Metformin alleviated oxidative stress and enhanced autophagy in diabetic kidney disease via AMPK/SIRT1-FOXO1 pathway	Ren et al. (2020)
AMPK signaling pathway	Fenofibrate	HFD induced C57BL/6J mice; PA induced mouse proximal tubule cells	Delayed treatment with fenofibrate had a therapeutic effect on HFD-induced kidney injury through the activation of AMPK and induction of subsequent downstream effectors: autophagy, fatty acid oxidation enzymes, and antioxidants	Sohn et al. (2017)
VEGF/PI3K/AKT/mTOR signaling pathway	Calcium dobesilate	HFD and STZ induced C57BL/6 mice; HFD induced KK-Ay mice	Calcium dobesilate played a key role in protecting renal function and restoring autophagy by blocking VEGF/VEGFR2 and inhibiting the PI3K/AKT/mTOR signaling pathway	Wang et al. (2019b)
AMPK-mediated signaling pathway	Metformin	High-glucose and high-fat diet and STZ induced SD rats; HG induced renal tubular epithelial cells	Metformin-induced AMPK significantly ameliorated renal autophagic function, inhibited the partial EMT of RTECs, and attenuated TIF, all of which effectively prevented or delayed the onset of DN.	Wang et al. (2021b)
FGF21 signaling pathway	Alprostadil (PGE1)	PA induced HK-2 cells	The researchers demonstrated the potential protection of PGE1 on insulin resistance in renal tubules via autophagy-dependent FGF21 pathway in preventing the progression of DN.	Wei et al. (2018)
AMPK/mTOR/NF-κB signaling pathway	Dapagliflozin	HG induced HK-2 cells	In diabetic renal proximal tubular cells, dapagliflozin ameliorated: HG-induced autophagic flux reduction, via increased AMPK activity and mTOR suppression; and inflammatory alterations due to NF-κB pathway suppression	Xu et al. (2021)
AMPK/eNOS signaling pathway	Liraglutide	SDT fatty rats	Liraglutide might exert a renoprotective effect via prevention of glomerular endothelial abnormality and preservation of autophagy in early-phase DKD, independent of blood glucose, and blood pressure levels	Yamada et al. (2021)
AMPK/mTOR signaling pathway	Liraglutide	ZDF rats; AGEs induced Ins 1 cells	The renoprotective effect of the GLP-1 in DKD rats and the underlying mechanism that was independent of controlling glucose were illustrated	Yang et al. (2020b)

DKD, diabetic kidney disease; AMPK, AMP-activated kinase; STZ, streptozotocin; HG, high glucose; mTOR, the mammalian target of rapamycin; P70S6K, ribosomal protein 70 S6 kinase; 4EBP1, 4E-binding protein 1; Ca-CAMKK2, calcium/calmodulin-dependent protein kinase kinase 2; HK-2, cells, human proximal tubular cells; VDR, Vitamin D receptor; SIRT1, sirtuin1; FOXO1, forkhead box transcription factor O1; HFD, high fat diet; SD, rats, Sprague-Dawley rats; RMCs, rat mesangial cells; PA, palmitic acid; VEGF, vascular endothelial growth factor; PI3K, phosphatidylinositol three kinase; AKT, protein kinase B; VEGFR2, VEGF, receptor 2; EMT, epithelial mesenchymal transition; RTECs, renal tubular epithelial cells; TIF, tubulointerstitial fibrosis; DN, diabetic nephropathy; FGF21, fibroblast growth factor 21; NF-κB, nuclear factor-kappaB; eNOS, endothelial nitric oxide synthase; SDT, fatty rats, spontaneously diabetic torii fatty rats; ZDF, rats, Zucker diabetic fatty rats; AGEs, advanced glycation end products; GLP-1, glucagon-like peptide 1.

**TABLE 2:** The role of autophagosome formation in DKD (drugs without clinical trials).

Signaling pathway	Processing method	Modeling method (animal/cell)	Conclusion	References
RAGE/mTOR signaling pathway	Paeoniflorin	AGEs induced HBZY-1 cells	Paeoniflorin inhibited AGEs induced autophagy and cell injury in HBZY-1 through activating mTOR and inhibiting RAGE.	Chen et al. (2017)
PI3K/Akt signaling pathway	Astilbin	HG induced HK-2 cells	Astilbin attenuated HG-induced autophagy and apoptosis in HK-2 cells through the PI3K/Akt pathway	Chen et al. (2018)
JAK/STAT signaling pathway	Ruxolitinib	STZ induced C57BL/6 J mice; HG induced MPC-5	JAK/STAT pathway promoted the progression of diabetic kidney disease via autophagy in podocytes	Chen et al. (2021a)
Beclin-1-Bcl-2 complex mediated signaling pathway	Carbon monoxide (CO)	HFD and STZ induced C57BL/6J mice; HG induced HBZY-1 cells, HK-2 cells and HPC.	CO protected DN mice from renal senescence and function loss via improving autophagy partly mediated by dissociating Beclin-1-Bcl-2 complex, which was possibly ascribed to the degradation of SASP.	Chen et al. (2021c)
ULK1-mediated signaling pathway	Geniposide	Unx, HFD and STZ induced C57BL/6 mice	Geniposide enhanced ULK1-mediated autophagy and reduced oxidative stress, inflammation, and fibrosis	Dusabimana et al. (2021)
AMPK/mTOR signaling pathway	Ethyl acetate	STZ induced Wistar albino rats	Phenolic-rich fraction had a protective effects against diabetic nephropathy presumably via enhancing autophagy and prevention of apoptosis	Ezzat et al. (2021)
AMPK-mediated signaling pathway	Astragaloside IV (AS-IV)	STZ induced C57BL/6J mice; HG induced podocytes	AS-IV prevented the progression of DN, which was mediated at least in part by SERCA2-dependent ER stress attenuation and AMPKα-promoted autophagy induction	Guo et al. (2017)
AdipoR1/AMPK signaling pathway	AdipoRon	DKD patients; db/db mice; HG induced HK-2 cells	Autophagy-mediated lipophagy deficiency played a critical role in the ectopic lipid deposition and lipid-related renal injury of DN.	Han et al. (2021)
AMPK signaling pathway	Dencichine	HFD and STZ induced SD rats; hydrogen peroxide induced HK-2 cells	Dencichine ameliorated renal injury by improving oxidative stress, apoptosis, and fibrosis in diabetic rats	Huang et al. (2020)
Signaling pathway	Processing method	Modeling method (animal/cell)	Conclusion	References
Smad1/mTOR signaling pathway	ADSCs-Exo	db/db mice; HG induced MPC-5	ADSCs-Exo vividly ameliorated DN symptom by enhancing the expression of miR-486 which led to the inhibition of Smad1/mTOR signaling pathway in podocyte	Jin et al. (2019)
PI3K/Akt/mTOR signaling pathway	Jiedu Tongluo Baoshen formula	HFD and STZ induced rats	Jiedu Tongluo Baoshen formula enhanced podocyte autophagy to reduce podocyte damage, thereby effectively treating DKD proteinuria and protecting kidney function	Jin et al. (2022)
NF-κB/iNOS signaling pathway	Valproic acid (VPA)	STZ induced SD rats	VPA treatment ameliorated the podocyte and renal injuries mainly by facilitating the autophagy and inactivation of NF-κB/iNOS signaling	Khan et al. (2015)
mTORC1 signaling pathway	Very-low-protein diet	Wistar fatty rats	A very-low-protein diet improved advanced diabetic renal injuries, including tubulointerstitial damage, by restoring autophagy through the suppression of the mTORC1 pathway	Kitada et al. (2016)
PI3K/Akt/NF-κB signaling pathway	Wogonin	STZ induced mice; HG induced HK-2 cells	Wogonin could mitigate tubulointerstitial fibrosis and renal tubular cell injury via regulating PI3K/Akt/NF-κB signaling pathway-mediated autophagy and inflammation	Lei et al. (2021)
miR-141-3p/PTEN/Akt/mTOR signaling pathway	Triptolide	HFD and STZ induced SD rats; HG induced human mesangial cells	Triptolide alleviated fibrosis by restoring autophagy through the miR-141-3p/PTEN/Akt/mTOR pathway	Li et al. (2017)
GSK3β signaling pathway	Sarsasapogenin (Sar)	STZ induced rats; HG induced podocytes	Sar meliorated experimental DN through targeting GSK3β signaling pathway and restoring podocyte autophagy	Li et al. (2021)
CaMKKβ-LKB1-AMPK signaling pathway	Cinacalcet	db/db mice; HG induced HGECs, and murine podocytes	Cinacalcet increased intracellular Ca2+ followed by an activation of CaMKKβ-LKB1-AMPK signaling in HGECs and podocytes in the kidney, and then modulated apoptosis and autophagy	Lim et al. (2018)
ERK/Akt/mTOR signaling pathway	Apelin	KKAy mice; HG induced podocytes	Increased apelin concentration in plasma inhibited podocyte autophagy, which would lead to podocyte apoptosis and renal dysfunction in diabetes	Liu et al. (2017)
AMPK signaling pathway	Cathelicidin-BF peptide	STZ induced rats; hydrogen peroxide induced HK-2 cells	Cathelicidin-BF attenuated kidney injury through inhibiting oxidative stress, inflammation, and fibrosis in streptozotocin-induced diabetic rats	Liu et al. (2020)
Bcl-2-mediated signaling pathway	Wogonin	STZ induced C57BL/6 J mice; HG induced MPC-5	Wogonin protected glomerular podocytes by targeting Bcl-2-mediated autophagy and apoptosis in diabetic kidney disease	Liu et al. (2022)
signaling pathway	Caffeic acid	HFD and STZ induced Wistar rats	Caffeic acid modulated autophagy pathway through inhibition of autophagy regulatory miRNAs, and then played a role in anti-diabetic nephropathy	Matboli et al. (2017)
AMPK-mTOR-ULK1 signaling pathway	Mangiferin	STZ induced SD rats	Mangiferin delayed the progression of DN and protected the podocytes by enhancing autophagy under diabetic conditions via the AMPK-mTOR-ULK1 pathway	Wang et al. (2018a)
SIRT1-NF-κB signaling pathway	AS-IV	KK-Ay mice; HG induced mesangial cells	AS-IV ameliorated renal function and morphology by inducing autophagy and inhibiting mesangial cells activation through the SIRT1-NF-κB pathway	Wang et al. (2018b)
SIRT-NF-κB P65 signaling pathway	AS-IV	KK-Ay mice; HG induced podocytes	AS-IV exerted its effects on podocyte EMT through modulation of the SIRT1-NF-κB pathway and autophagy activation	Wang et al. (2019a)
GPER/Keap1/Nrf2 signaling pathway	Icariin	STZ induced SD rats; HG induced human mesangial cells	The therapeutic effects of icariin on type 1 diabetic nephropathy were demonstrated in rats via GPER mediated P62-dependent Keap1 degradation and Nrf2 activation	Wang et al. (2020)
Nrf2/PINK signaling pathway	MitoQ (mitochondria-targeted antioxidant)	db/db mice; HG induced HK-2 cells	MitoQ exerted beneficial effects on tubular injury in DKD via mitophagy and that mitochondrial quality control was mediated by Nrf2/PINK.	Xiao et al. (2017)
PI3K/AKT/mTOR signaling pathway	Paecilomyces cicadae-fermented Radix astragali (RPF)	HFD and STZ induced C57BL/6 mice; HG induced podocytes	RPF enhanced autophagy in podocytes and delayed DN probably by inhibiting the PI3K/AKT/mTOR signaling pathway	Yang et al. (2020a)
AMPK/mTOR signaling pathway	Erlotinib	STZ induced C57BLKS/J mice (eNOS−/− mice)	Inhibition of EGFR with erlotinib attenuated the development of diabetic nephropathy in type 1 diabetes, which was mediated at least in part by inhibition of mTOR and activation of AMPK, with increased autophagy and inhibition of ER stress	Zhang et al. (2014)
AMPK-mTOR signaling pathway	Cyclocarya paliurus triterpenic acids (CPT)	STZ induced SD rats; HG induced HK-2 cells	CPT might be a desired candidate against diabetes, potentially through AMPK-mTOR-regulated autophagy pathway	Zhang et al. (2019)
AMPK-mediated signaling pathway	Huang-Gui Solid Dispersion (HGSD)	HFD and STZ induced Wistar rats, db/db mice	HGSD protected against diabetic kidney dysfunction by inhibiting glomerular mesangial matrix expansion and activating autophagy	Zhang et al. (2020)
AMPK/mTOR signaling pathway	Spermine	STZ induced Wistar rats; HG induced podocytes	Spermine might have the potential to prevent diabetic kidney injury in rats by promoting autophagy via regulating the AMPK/mTOR signaling pathway	Zhang et al. (2021)
CaMKK2-AMPK-p-mTOR signaling pathway	Jujuboside A (JuA)	HFD and STZ induced SD rats	JuA protected against type II diabetic nephropathy through inhibiting oxidative stress and apoptosis mediated by mitochondria and ER stress. In addition, autophagy and mitophagy were enhanced by JuA	Zhong et al. (2022)

RAGE, the receptor for advanced glycation end-products; HBZY-1, cells, rat mesangial cells; JAK/STAT, the Janus kinase (JAK)/signal transductors and the transcription (STAT); MPC-5, murine podocyte MPC-5, cells; Beclin-1, mammalian ortholog of the yeast autophagy-related gene 6 (Atg6) and BEC-1, in the C.*elegans* nematode; Bcl-2, B cell lymphoma-2; SASP, senescence-related secretory phenotype; Unx, unilateral nephrectomy; ULK1, unc-51-like kinase 1; ER, endoplasmic reticulum; SERCA2, sarcoplasmic/endoplasmic reticulum Ca(2^+^) ATPase, 2; AdipoR1, adiponectin receptor 1; Smad1, Smad family member 1; ADSCs, adipose-derived stem cells; ADSCs-Exo, ADSCs-derived exosome; iNOS, inducible nitric oxide synthase; mTORC1, the mechanistic target of rapamycin complex 1; PTEN, phosphatase and tensin homolog; GSK3β, glycogen synthase kinase-3β; LKB1, liver kinase B1; HGECs, human glomerular endothelial cells; ERK, extracellular signal-regulated kinase; GPER, G-protein-coupled estrogen receptor; Keap1/Nrf2, the Kelch-like ECH-associated protein 1/nuclear factor erythroid 2 related factor 2; PINK, tensin homolog (PTEN)-induced putative kinase; EGFR, epidermal growth factor receptor.

**TABLE 3:** The role of autophagosome formation in DKD (low or high expression of a certain target).

Signaling pathway	Processing method	Modeling method (animal/cell)	Conclusion	References
Akt/mTOR signaling pathway	LncRNA SOX2OT overexpression	STZ induced C57BL/6 mice; HG induced mouse mesangial cells	LncRNA SOX2OT alleviated the pathogenesis of DN via regulating Akt/mTOR-mediated autophagy	Chen et al. (2021b)
AKT/SIRT-1/FOXO3a signaling pathway	P2Y2R knockout	Unx, HFD and STZ induced C57BL/6 mice	P2Y2R contributed to the pathogenesis of DN by impairing autophagy and served as a therapeutic target for treating DN.	Dusabimana et al. (2020)
FOXO1/STAT1 signaling pathway	Loss of TIMP3	TIMP3(−/−) mice; HG induced mesangial cells	Reduction of TIMP3 caused a concomitant STAT1-dependent and compartment-specific loss of FOXO1 activity, which in turn diminished the expression of protective autophagy genes to fuel glomeruli damage in a mouse model	Fiorentino et al. (2013)
mTOR/S6K signaling pathway	KLF4 overexpression	db/db mice; DKD mice serum induced podocytes	KLF4 ameliorated DKD by activating autophagy via the mTOR pathway	Gong et al. (2019)
mTOR signaling pathway	Reduce the expression of TXNIP	DKD patients; STZ induced rats; HG induced HK-2 cells	Hyperglycemia-induced TXNIP contributed to the dysregulation of tubular autophagy and mitophagy in DN through activation of the mTOR signaling pathway	Huang et al. (2016)
ERK/EGR1 signaling pathway	Gene knockdown of GPR43	HG induced podocytes	GPR43 activation-mediated lipotoxicity contributed to podocyte injury in DN by modulating the ERK/EGR1 pathway	Lu et al. (2022)
P53/miR-214/ULK1 signaling pathway	LNA–anti–miR-214	STZ induced C57BL/6J and Akita mice; HG induced proximal tubule cells	Autophagy impairment was instigated by the downregulation of ULK1 via P53-mediated induction of miR-214	Ma et al. (2020)
HDAC4-STAT1 signaling pathway	pGLV3-shRNA2-HDAC4	Unx and STZ induced SD rats, db/db mice; HG, AGEs or TGF-β induced podocytes	HDAC4 contributed to podocyte injury and was one of critical components of a signal transduction pathway that linked renal injury to autophagy in diabetic nephropathy	Wang et al. (2014)
P53/miR-155-5p/SIRT1 signaling pathway	Change the expression of miR-155 and SIRT1	HG induced HK-2 cells	The signaling axis of P53, miR-155-5p, and SIRT1 in autophagic process might be a critical adapting mechanism for diabetic kidney injury	Wang et al. (2018c)
SPAG5/AKT/mTOR signaling pathway	Silencing SPAG5	HG induced human podocytes	SPAG5 antisense RNA1 inhibited autophagy and aggravated apoptosis of podocytes via SPAG5/AKT/mTOR pathway	Xu et al. (2020)
mTOR/TFEB signaling pathway	Inhibit the activity of mTOR and TFEB	db/db mice; AGEs induced podocytes	AGEs inhibited the formation and turnover of autophagosomes in podocytes by activating mTOR and inhibiting the nuclear translocation of TFEB.	Zhao et al. (2018a)
EGR1 signaling pathway signaling pathway	CIDEC silencing	HFD and STZ induced SD rats; HG induced NRK-52E cells	CIDEC gene silencing might delay the progression of DN by restoring autophagy activity and inhibiting apoptosis with the participation of EGR1and ATGL.	Zheng et al. (2021)

LncRNA SOX2OT, lncRNA SOX2 overlapping transcript; P2Y2R, purinergic receptors; TIMP3, tissue inhibitor of metalloproteinase three; KLF4, Krüppel-like factor 4; TXNIP, thioredoxin-interacting protein; EGR1, early growth response factor 1; GPR43, G-protein-coupled receptor 43; HDAC4, histone deacetylase 4; TGF-β, transforming growth factor-beta; SPAG5, sperm-associated antigen 5; TFEB, transcription factor EB; CIDEC, cell death-inducing DFF45-like effector C; NRK-52E, renal tubular epithelial cell.

#### 4.1.2 Role of autophagy in renal tubular epithelial cells

In DKD mice and renal tubular epithelial cell models, treatment with LNA-anti-miR-214 or overexpression of Klotho (an anti-aging factor expressed in the kidney) modulated the P53/miR-214/ULK1 and AMPK/ERK signaling pathways to exert nephroprotective effects (Ma et al., 2020; Xue et al., 2021). In a different study model, Korean red ginseng was shown to promote autophagy and thus reduced hyperglycaemia-induced renal inflammation and fibrosis, exerting a nephroprotective effect (Karunasagara et al., 2020). In a study using high glucose induced human proximal tubular cells (HK-2 cells) as a model, the investigators found that renal injury could be ameliorated by altering the expression of miR-155 and SIRT1, or by administering dapagliflozin and astilbin, respectively (Chen et al., 2018; Wang Y. et al., 2018; Xu et al., 2021). However, the difference between these two drugs was that dapagliflozin acted to suppress inflammation by regulating the AMPK/mTOR/NF-κB pathway, whereas astilbin worked on the PI3K/AKT signaling pathway to attenuate high glucose-induced autophagy and apoptosis (Chen et al., 2018; Xu et al., 2021). Carbon monoxide positively regulated autophagy via the Beclin-1-Bcl-2 pathway in the DKD model to protect the kidney from injury and aging; meanwhile, wogonin attenuated renal fibrosis by targeting PI3K/AKT/NF-κB to regulate renal inflammation and autophagic activity (Chen L. et al., 2021; Lei et al., 2021). In palmitic acid-induced HK-2 cells, alprostadil ameliorated renal tubular insulin resistance and restored autophagy via an autophagy-dependent fibroblast growth factor 21-associated pathway (Wei et al., 2018). Cyclocarya paliurus triterpenic acids or reduced expression of thioredoxin interacting protein (TXNIP) acted through mTOR or in combination with the AMPK pathway to induce autophagy (Huang et al., 2016; Zhang et al., 2019). In contrast, the Chinese herbal extract icariin from Epimedium restored autophagy and attenuated renal tubulointerstitial fibrosis by targeting miR-192-5p/GLP-1R (GLP-1 receptor) in a DKD model (Jia et al., 2021). At the same time, the overexpression of TXNIP (one of the factors of autophagy) induced by high glucose led to autophagy dysfunction and participated in the occurrence of DKD (Huang et al., 2014). Studies had shown that the drug empagliflozin or paricalcitol (an activated vitamin D analog) restored autophagy via AMPK-related pathways in DKD models (Lee et al., 2019; Li et al., 2022). Treatment with dencichine or Cathelicidin-BF peptide in DKD rats and cellular models showed that both positively regulated autophagy through an AMPK-mediated pathway and improved renal function by reducing oxidative stress and fibrosis (Huang et al., 2020; Liu et al., 2020). However, experiments in which metformin treated DKD rats and renal tubular epithelial cells models, metformin exerted therapeutic effects through an AMPK-mediated pathway (Wang F. et al., 2021). To investigate the specific mechanism of fenofibrate on the DKD model, researchers found that fenofibrate induced autophagy and acted as an antioxidant via the AMPK pathway (Sohn et al., 2017). In addition, researchers have experimentally demonstrated that the use of therapeutic agents and approaches such as mitoQ (mitochondria-targeted antioxidant) and NSC697923 inhibitor and t-AUCB (an inhibitor of soluble epoxide hydrolase) can slow down the progression of DKD by improving autophagy (Xiao et al., 2017; Pontrelli et al., 2018; Jiang et al., 2020; Han et al., 2021; Liang et al., 2021; Zheng et al., 2021). In summary, autophagy plays an important role in maintaining the homeostasis and function of renal tubular epithelial cells. If autophagy is imbalanced, it leads to intracellular metabolic changes and accelerates the onset and progression of disease, but these could be restored with drugs such as metformin and empagliflozin (Figure 2; Table.1-3, Table.5).

#### 4.1.3 Role of autophagy in glomerular mesangial cells

Treatment of induced rat mesangial cells and animals with metformin and paeoniflorin showed that metformin alleviated oxidative stress and enhanced autophagy via the AMPK/SIRT1-FOXO1 pathway (Ren et al., 2020). In contrast, paeoniflorin reduced LC3II/LC3I expression through the RAGE/mTOR pathway, decreased the number of autophagosomes and inhibited AGEs-induced autophagy (Chen et al., 2017). To investigate the role of lncRNA SOX2OT and tissue inhibitor of metalloproteinase 3 (TIMP3) in the progression of DKD, overexpression or gene deletion was used in treated mice and mouse mesangial cells, suggesting that the overexpression of lncRNA SOX2OT (SOX2 overlapping transcript) restored autophagy via AKT/mTOR, while TIMP3 deletion led to reduced FOXO1 expression of the autophagy key factor and increased expression of the FOXO1 transcriptional repressor STAT1, driving renal function to deteriorate in DKD patients and mice (Fiorentino et al., 2013; Chen K. et al., 2021). In addition to this, AS-IV induced autophagy and inhibited activation of mesangial cells in the SIRT1-NF-κB pathway, alleviating mesangial matrix expansion and proliferation in the KK-Ay mouse model (Wang et al., 2018b). In DKD rat and HMC models, icariin promoted G-protein-coupled estrogen receptor-mediated P62-dependent Keap1 degradation as well as Nrf2 activation, and played a therapeutic role in the progression of type 1 DKD in rats (Wang et al., 2020). Similarly, triptolide targeted the miR-141-3p/PTEN/AKT/mTOR pathway to induce autophagy and protect renal function (Li et al., 2017). By summarizing the above literature, we found that autophagy exists in glomerular mesangial cells. Oxidative stress and inflammation inhibit autophagy in mesangial cells, but the application of drugs such as AS-IV and Icariin can upregulate the occurrence of autophagy by regulating autophagy-related pathways (Figure 2; Tables.1-3, Table.5).

#### 4.1.4 Other studies on autophagosome formation in DKD

The use of GLP-1 in Zucker diabetic fatty rats and AGEs induced Ins 1 cells model showed that GLP-1 showed renoprotective effects independent of hypoglycaemia (Yang S. et al., 2020). Using liraglutide in an animal model of spontaneously diabetic tori fatty rats, it was concluded that liraglutide protected the kidney and activated autophagy in early DKD via the AMPK/eNOS pathway independent of reductions in blood glucose and blood pressure (Yamada et al., 2021). Caffeic acid, ethyl acetate or Huang-Gui Solid Dispersion (HGSD) was used in a model of Wistar rats with established DM, and it was ultimately concluded that all three drugs could activate autophagy through AMPK-mediated pathways, switching on of key downstream genes (Matboli et al., 2017; Zhang et al., 2020; Ezzat et al., 2021). Purinergic receptors P2Y2R knockout impaired autophagy via the AKT/SIRT-1/FOXO3a signaling pathway in an experiment using unilateral nephrectomy combined with high fat diet and STZ (Dusabimana et al., 2020). However, in two other studies using the same animal model, the use of the drug geniposide or Abelmoschus manihot activated autophagy, reduced fibrosis and inflammation, as a result slowed the progression of DKD (Kim et al., 2018; Dusabimana et al., 2021). Meanwhile, erlotinib, Cordyceps militaris polysaccharides, and calcium dobesilate were used in STZ-induced mice as a model to demonstrate that erlotinib activated AMPK, inhibited mTOR activity and thus epidermal growth factor receptor, and played a role in the renal protective mechanism (Zhang et al., 2014). And another drug calcium dobesilate restored autophagy by blocking the VEGF/PI3K/AKT/mTOR cascade reaction and exerted nephroprotective effects together with Cordyceps militaris polysaccharides (Wang et al., 2019b; Chen et al., 2019). In a DKD rat model, mangiferin increased the number of autophagosomes in DKD rat podocytes and reduce proteinuria in diabetic rats, and this result was achieved by upregulating phosphorylated AMPK and ULK1 as well as downregulating mTOR phosphorylation (Wang et al., 2018a). Similarly, in a study on jujuboside A (JuA), it was demonstrated that JuA also promoted autophagy and mitophagy by activating CaMKK2, an upstream regulator of AMPK, alleviating the hyperglycaemic state and reducing various indicators of renal function (Zhong et al., 2022). The Chinese herbal formula Jiedu Tongluo Baoshen formula and valproate could counteract renal injury by modulating PI3K/AKT/mTOR or NF-κB/iNOS pathways, respectively (Khan et al., 2015; Jin et al., 2022). The use of paricalcitol (vitamin D receptor agonist) improved autophagic fluxes and improved slit diaphragm-tight junction conversion in DKD rats and db/db mice (Wang B. et al., 2021). A study on the protective effects of isorhamnetin in DKD rats had also demonstrated that isorhamnetin had good hypoglycaemic and hypolipidaemic properties, increased the number of autophagosomes and had a nephroprotective effect (Matboli et al., 2021). Finally, very low protein diets restored autophagy by inhibiting mTOR-related pathways (Kitada et al., 2016). In addition, by consulting the literatures, we also know that IL-17A and SCO-792 (an enteropeptidase inhibitor) improved DKD by regulating autophagy (Kim et al., 2021; Sugama et al., 2021). In a conclusion, in experiments using only animals as research models, researchers have found that drugs can activate autophagy, regulate autophagy-related pathways and key autophagy factors, and exert nephroprotective effects (Figure 2; Table.1-3, Table.5).

**TABLE 5:** The role of autophagosome formation in DKD (supplementary material).

Signaling pathway	Processing method	Modeling method (animal/cell)	Conclusion	References
-	Ursodeoxycholic acid (UDCA)and 4-phenylbutyrate (4-PBA)	db/db mice; HG induced podocytes	UDCA and 4-PBA prevented HG-induced podocyte apoptosis by alleviating ER stress and restoring autophagy	Cao et al. (2016)
-	Cordyceps militaris polysaccharides (CMP)	STZ induced C57BL/6 mice	CMP exerted a protective effect on DN in STZ-treated mice possibly via activation of autophagy	Chen et al. (2019)
TXNIP- mediated signaling pathway	Reduce TXNIP overexpression	STZ induced C57B/6 mice; HG induced proximal tubular cells	HG-induced overexpression of TXNIP might contribute to the dysfunction of tubular autophagy in diabetes	Huang et al. (2014)
miR-383-5p-mediated signaling pathway	Resveratrol	db/db mice; HG induced podocytes	Resveratrol effectively attenuated high glucose-induced apoptosis via the activation of autophagy in db/db mice and podocytes, which involved miR-383-5p	Huang et al. (2017)
-	Protein tyrosine phosphatase 1B(PTP1B)disruption	HFD and STZ induced C57BL/6J mice (Ptpn1fl/fl mice); HG induced podocytes	Podocyte PTP1B deficiency attenuated hyperglycemia-induced renal damage	Ito et al. (2017)
miR-192-5p/GLP-1R signaling pathway	Icariin	High-sugar and high-fat diet and STZ induced SD rats; HG induced HK-2 and NRK-49F cells	Icariin alleviated tubulointerstitial fibrosis by restoring autophagy through the miR-192-5p/GLP-1R pathway	Jia et al. (2021)
-	t-AUCB (an inhibitor of sEH)	db/db mice; HG induced HK-2 cells	t-AUCB played a protective role in hyperglycemia-induced proximal tubular injury and that the potential mechanism of t-AUCB-mediated protective autophagy was involved in modulating mitochondrial function and ER stress	Jiang et al. (2020)
-	Korean red ginseng	STZ induced SD rats; HG induced HK-2 cells	Korean red ginseng attenuated hyperglycemia-induced renal inflammation and fibrosis via accelerated autophagy and protected against diabetic kidney disease	Karunasagara et al. (2020)
-	Abelmoschus manihot	Unx, HFD and STZ induced C57BL/6 mice	Supplementation of Abelmoschus manihot ameliorated diabetic nephropathy and hepatic steatosis by activating autophagy in mice	Kim et al. (2018)
-	IL-17A knockout	STZ induced C57BL/6 mice (IL-17A KO mice)	IL-17 deficiency aggravated of STZ-induced DN via attenuation of autophagic response	Kim et al. (2021)
ATF4-mediated signaling pathway	Inhibit the expression of ATF4	STZ induced ATF4 ± heterozygous mice; HG induced NRK-52E cells	ATF4 promoted renal tubulointerstitial fibrosis by suppressing autophagy in diabetic nephropathy	Liang et al. (2021)
-	Isorhamnetin	HFD and STZ induced Wistar rats	Isorhamnetin improved fasting blood glucose, renal and lipid profiles with increased autophagosomes in renal tissues, and suppressed miRNA regulation of autophagy genes, finally played a renal protective role	Matboli et al. (2021)
-	NSC697923 inhibitor	DN Patients; HG induced HK-2 cells	Prolonged hyperglycemia in diabetic patients could impair autophagy as a consequence of Lys63-Ub protein accumulation, thus promoting intracellular autophagic vesicles increase, finally leading to tubular cell death in DN.	Pontrelli et al. (2018)
-	SCO-792 (enteropeptidase inhibitor)	WF rats	SCO-792-induced therapeutic efficacy was likely to be independent of glycaemic control and mediated by the regulation of AAs and autophagy, so as to improve the condition of DKD patients	Sugama et al. (2021)
VDR/Atg3 signaling pathway	Paricalcitol (Vitamin D receptor agonist (VDRA))	STZ induced SD rats; db/db mice	VDR/Atg3 axis deficiency resulted in SD-TJ transition and foot processes effacement via blocking the P62-mediated autophagy pathway in DN.	Wang et al. (2021a)
HO-1-mediated signaling pathway	Celastrol	HG induced podocytes	Celastrol might protect against HG-induced podocyte injury, inflammation and insulin resistance by restoring HO-1-mediated autophagy pathway	Zhan et al. (2018)

GLP-1R, GLP-1, receptor; NRK-49F, rat kidney fibroblasts; sEH, soluble epoxide hydrolase; IL-17A, interleukin 17A; Lys63-Ub, lysine 63 ubiquitination; WF, rats, Wistar fatty rats; SD-TJ, slit diaphragm-tight junction; HO-1, heme oxygenase.

### 4.2 The role of autolysosome formation in DKD

In experiments with DKD patients and AGEs-induced podocytes, researchers found that AGEs did not induce autophagy in podocytes, but increased LC3-II and P62-positive vacuoles, demonstrating that the autophagic degradation pathway was disrupted. This was caused by increased lysosomal membrane permeability inducing lysosomal dysfunction; however, therapeutic measures of resveratrol plus vitamin E could improve this situation (Liu et al., 2019). In db/db mice and AGE-BSA-induced HK-2 cells, restoration of Smad3 expression promoted TFEB-dependent lysosomogenesis (Yang C. et al., 2021). AGEs was able to induce an increase in the number of autophagosomes, the accumulation of sequestosome 1 protein and a decrease in the number of autolysosomes in AGEs-induced HK-2 cells. Therefore, this experiment concluded that AGEs was able to cause inactivation of autophagy, accelerate increased lysosomal permeability and dysfunction in HK-2 cells (Liu et al., 2015). Hepatocyte growth factor was used on high glucose-induced MPC-5 and STZ-induced mice, which regulated the PI3K/AKT-GSK3β-TFEB signaling pathway, improved lysosomal function and increased autophagic activity (Hou et al., 2020). In the ATG5 knockout mouse model and proximal tubular epithelial cells model, defective autophagy downregulated lysosomal biogenesis and promote the accumulation of AGEs, leading to DKD progression (Takahashi et al., 2017). In a study using STZ-induced Sprague-Dawley rats after high-glucose and high-fat model, the drug Keluoxin improved biochemical indicators, reduce podocyte damage and modulate lysosomal function in DKD rats, mitigating the progression of DKD (Yang X. et al., 2021). In a separate study, a group of STZ-induced male BALB/c mice were injected with mesenchymal stem cells (MSCs) via tail vein while primary MSCs were extracted from BALB/c mice for culture and then transfected with TFEB RNA. The results demonstrated that MSCs shifted the M2 phenotype from M1/M2 to macrophages and produce a nephroprotective effect, which was mediated by TFEB (Yuan Y. et al., 2020). Meanwhile, a study on the mechanism of high doses of vitamin E treatment with DKD had shown that high doses of vitamin E could promote lysosomal function and reduce autophagic stress to delay the progression of DKD (Zhao Y. et al., 2018). In addition to this, in order to investigate the role of the vaspin-HSPA1L (heat shock protein family A member 1 like) complex in the development of DKD, the present study made use of patients with obesity-related nephropathy and DKD as well as high-fat and high-glucose-induced mice as models, and then concluded that the vapsin/HSPA1L-mediated pathway played a role in ER stress, impaire autophagy and lysosomal function (Nakatsuka et al., 2021). In summary, the formation of the autolysosome is another key pathway in the autophagic process and is related to the degradation of intracellular metabolites (Wu M. et al., 2021). Excessive production of AGEs or knockdown of key autophagy factors leads to increased lysosomal membrane permeability or downregulated lysosomal biogenesis and impaired lysosomal function, resulting in disruption of the autophagic degradation pathway (Levy et al., 2017; Liu et al., 2019). However, therapeutic measures such as resveratrol plus vitamin E can promote autophagy and slow down the progression of DKD in terms of restoring lysosomal function (Figure 2; Table.4).

**TABLE 4:** The role of autolysosome formation in DKD.

Signaling pathway	Processing method	Modeling method (animal/cell)	Conclusion	References
PI3K/Akt-GSK3β-TFEB signaling pathway	Hepatocyte growth factor (HGF)	STZ induced DBA/2J mice; HG induced MPC-5	HGF protected against diabetic nephropathy through restoring podocyte autophagy, which at least partially involved PI3K/Akt-GSK3β-TFEB axis-mediated lysosomal function improvement	Hou et al. (2020)
-	-	DKD patients; AGEs induced HK-2 cells	Lysosomal membrane permeabilization and lysosomal dysfunction were triggered by AGEs, which induced autophagic inactivation in renal tubular epithelial cells from patients with DN.	Liu et al. (2015)
-	Resveratrol plus vitamin E	DKD patients; AGEs induced podocytes	Lysosomal membrane permeabilization induced lysosomal dysfunction, which was a key node of insufficient autophagy of podocytes in DKD.	Liu et al. (2019)
Vaspin/HSPA1L-mediated signaling pathway	Vaspin knockout	Obesity related kidney disease and DN patients; high fat-high sucrose diet induced C57BL/6J mice	Vaspin maintained PTCs through ameliorating ER stress, autophagy impairment, and lysosome dysfunction in DKD via vapsin/HSPA1L-mediated pathways	Nakatsuka et al. (2021)
TFEB-mediated signaling pathway	ATG5 knockout	STZ induced Atg5F/F KAP mice; HG induced PTECs	Autophagy inhibited the accumulation of AGEs by promoting lysosomal biogenesis and function in the kidney proximal tubules	Takahashi et al. (2017)
Smad3-mediated signaling pathway	Inhibit the expression of Smad3	db/db mice; AGE-BSA induced HK-2 cells	Smad3 promoted lysosome depletion via the inhibition of TFEB-dependent lysosome biogenesis	Yang et al. (2021a)
-	Keluoxin	High-fat and high-sugar diet and STZ induced SD rats	Keluoxin could regulate autophagy via improving the lysosomal degradation function and alleviating podocyte injury, alleviated kidney injury in rats with DN, and had a protective effect on renal function	Yang et al. (2021b)
TFEB-mediated signaling pathway	MSCs injection and TFEB RNAi transfection	STZ induced BALB/c mice; cultured primary MSCs	Mesenchymal stem cells restored lysosomal function and activated autophagy, and then suppressed inflammatory response and alleviated renal injuries in DN mice via TFEB-dependent Mφ switch	Yuan et al. (2020b)
-	High dose vitamin E	DN patients; STZ induced SD rats; AGE-BSA induced HK-2 cells	High dose vitamin E attenuated DN via improvement of lysosomal function and alleviation of autophagic stress	Zhao et al. (2018b)

HSPA1L, heat shock protein family A member 1 like; PTCs, proximal tubular cells; ATG, autophagy-related genes; PTECs, proximal tubular epithelial cells; MSCs, mesenchymal stem cells.

## 5. Discussion

Defective autophagy is present in the pathophysiology of many diseases and has been studied in the fields of oncology, neurodegenerative diseases, metabolic diseases and renaldiseases, where it is closely related to the pathological process of DKD (Mizushima and Levine, 2020).

AMPK activation by upstream kinases rapidly phosphorylates the ULK-1 complex while inhibiting the activation of mTOR (Alers et al., 2012). Subsequent phosphorylation of downstream Beclin-1 activates the PI3K complex, and promotes the synthesis of PI3P (Axe et al., 2008; Russell et al., 2013; Baskaran et al., 2014). Then PI3P recruits autophagy factors, inducing the formation of omegasomes which work as the initial part of the phagophore (Lamb et al., 2013). Subsequently, omegasomes are continuously extended by the action of DCFP1 and WIPI (Lamb et al., 2013). In parallel, two downstream ubiquitin complexes (ATG12-ATG5-ATG16L1 and LC3-II) are formed and then recruited to the phagophore upon induction of WIPI and ATG16L1 (Sou et al., 2006; Dooley et al., 2014; Popelka et al., 2021). Subsequently, the autophagosome wrapped around the cargo fuses with the lysosome and is regulated by the VPS34 complex II and the HOPS complex as well as Rab7 (Bröcker et al., 2012; Lamb et al., 2013; Pu et al., 2016). It is eventually degraded by the lysosome and excreted from the cell in the form of exocytosis for subsequent recycling and use (Buratta et al., 2020).

Hyperglycaemia stimulates the production of metabolites such as ROS and AGEs, which promote oxidative stress, inflammation and impair autophagy, while drugs can slow the progression of DKD by inducing or restoring autophagy (Zheng et al., 2020; Winiarska et al., 2021). In recent years relevant reports have discussed drugs that target autophagy in the treatment of DKD, such as the herbal extracts, the emerging GLP-1 activators and SGLT2 inhibitors and so on, all of which have been shown to modulate the autophagic process and improve renal function (Huang et al., 2017; Wang et al., 2018b; Winiarska et al., 2021; Yamada et al., 2021).

Autophagy acts as a reactive protective mechanism in the kidney in order to protect cells from stressors, and mTOR is generally inhibited in this situation (Kim et al., 2011). However, the inhibition of autophagy that results from mTOR activation does not imply cell damage, but rather promotes cell proliferation and contributes to kidney repair (Xie et al., 2015; Viana et al., 2018). Similarly, in a separate study, autophagy was found to have a possible role in promoting renal fibrosis: in severe acute kidney injury, tubular cells undergo arrest in the G2/M phase, leading to the secretion of pro-fibrotic factors that promotes the progression of renal fibrosis, accompanied by the emergence of TOR-autophagic spatially coupled compartments structures (distinct cytoplasmic compartments rich in mTORC1) (Yang et al., 2010; Canaud et al., 2019; Tang et al., 2020). In addition, side effects associated with the mTOR inhibitor rapamycin for DKD, such as hyperglycaemia, insulin resistance and dyslipidaemia, have also been reported, which may be related to the metabolic profile of the pancreas and other insulin-resistant tissues, including the liver and muscle (Viana et al., 2018). Therefore, the existence of autophagy is therefore like a double-edged sword.

However, there are many questions about autophagy that have not been clearly described. Three types of autophagy are known, namely microautophagy, macroautophagy and chaperone-mediated autophagy, and have been described and studied in details (Li et al., 2012; Feng et al., 2014; Dash et al., 2019). However, it is not clear whether there is an interconnection among the three of them, and the specific triggering mechanism of autophagy in them is also lacking. Further discoveries are needed about the specific mechanisms by which autophagy produces stress responding to human metabolites. On the other hand, there are no extensive experimental studies on the exact mechanism of autophagosome-lysosome fusion regulation and degradation and a large body of evidence is lacking to support this view. In addition, the exact mechanism of action of drugs targeting autophagy is still unclear, which has prevented the translation of some drugs from animal studies to human clinical studies for the time being, and therefore lacks theoretical support for human clinical studies. We have now affirmed the positive role of autophagy for the treatment of DKD and it can be one of the key directions for the future treatment of DKD (Ding and Choi, 2015). However, autophagy also has side effects, so how to manipulate autophagy is an important issue for researchers today. The above issues will need to be refined over time with further research.

## 6 Conclusion

Activation of autophagy protects renal function, while impaired autophagy is involved in DKD progression. Although drugs targeting autophagy are still being investigated and some have already been used clinically, the incidence of DKD is still on the rise, so maintaining autophagy in the kidney’s intrinsic cells is now an urgent issue in the medical field. By reviewing the physiological process of autophagy and the roles of DKD therapeutic agents as well as related pathways, we hope to provide new perspectives and inspiration for the clinical treatment of DKD.
